# Respiratory ß-2-Microglobulin exerts pH dependent antimicrobial activity

**DOI:** 10.1080/21505594.2020.1831367

**Published:** 2020-10-22

**Authors:** Armin Holch, Richard Bauer, Lia-Raluca Olari, Armando A. Rodriguez, Ludger Ständker, Nico Preising, Merve Karacan, Sebastian Wiese, Paul Walther, Yasser B. Ruiz-Blanco, Elsa Sanchez-Garcia, Christian Schumann, Jan Münch, Barbara Spellerberg

**Affiliations:** a Institute of Medical Microbiology and Hygiene, University Hospital, Ulm, Germany; b Institute of Molecular Virology, University Hospital, Ulm, Germany; c Core Facility Functional Peptidomics, Ulm University Medical Center, Ulm, Germany; d Core Unit Mass Spectrometry and Proteomics, Ulm University, Ulm, Germany; e Central Facility for Electron Microscopy, Ulm University Medical Center, Ulm, Germany; f Computational Biochemistry, Center of Medical Biotechnology, University of Duisburg-Essen, Essen, Germany; g Pneumology, Thoracic Oncology, Sleep and Respiratory Critical Care Medicine, Clinics Kempten-Allgäu, Kempten and Immenstadt, Germany

**Keywords:** ß2-microglobulin, antimicrobial peptides, innate immunity, pH, amyloid

## Abstract

The respiratory tract is a major entry site for microbial pathogens. To combat bacterial infections, the immune system has various defense mechanisms at its disposal, including antimicrobial peptides (AMPs). To search for novel AMPs from the respiratory tract, a peptide library from human broncho-alveolar-lavage (BAL) fluid was screened for antimicrobial activity by radial diffusion assays allowing the efficient detection of antibacterial activity within a small sample size. After repeated testing-cycles and subsequent purification, we identified ß-2-microglobulin (B2M) in antibacterially active fractions. B2M belongs to the MHC-1 receptor complex present at the surface of nucleated cells. It is known to inhibit the growth of *Listeria monocytogenes* and *Escherichia coli* and to facilitate phagocytosis of *Staphylococcus aureus*. Using commercially available B2M we confirmed a dose-dependent inhibition of *Pseudomonas aeruginosa* and *L. monocytogenes*. To characterize AMP activity within the B2M sequence, peptide fragments of the molecule were tested for antimicrobial activity. Activity could be localized to the C-terminal part of B2M. Investigating pH dependency of the antimicrobial activity of B2M demonstrated an increased activity at pH values of 5.5 and below, a hallmark of infection and inflammation. Sytox green uptake into bacterial cells following the exposure to B2M was determined and revealed a pH-dependent loss of bacterial membrane integrity. TEM analysis showed areas of disrupted bacterial membranes in *L. monocytogenes* incubated with B2M and high amounts of lysed bacterial cells. In conclusion, B2M as part of a ubiquitous cell surface complex may represent a potent antimicrobial agent by interfering with bacterial membrane integrity.

## Introduction

The respiratory tract represents one of the major entry sites for microbial pathogens. While ventilation and alveolar gas exchange ensures a proper oxygen supply of the human body it also exposes the mucosal membranes of the upper and lower respiratory tract to infectious agents and renders them into a predilection site for infections. Various microbial pathogens affect the lower respiratory tract causing serious and often life-threatening pulmonary infections. Lower respiratory tract infections (LRTI) have major health implications and represented in 2015 the fifth leading cause of death globally and the leading cause of death in children younger than 5 years [1]. To prevent these types of infection the respiratory tract is equipped with various defense mechanisms. Secreted mucus acts as protective layer that binds inhaled noxious substances and pathogens. Kinocilia, of the respiratory epithelium, create a tracheo-oral flow of mucus to remove bacteria and other substances from the airways, a mechanism known as mucociliary clearance. Additionally, the innate immune system offers protection against respiratory infections through antimicrobial peptides (AMPs). These peptides comprise a large group of anti-infective agents with anti-bacterial [2], anti-viral [3] or anti-fungal [4] activity. Further functions may include immunomodulatory properties. Most AMPs are small cationic peptides (10–50 amino acids) with amphiphilic properties, facilitating the insertion of AMPs into the lipid bilayer of bacterial cell membranes. The well-known AMPs LL37, belonging to the cathelicidin family and human beta-defensins have previously been shown to be present in the respiratory tract [5,6].

Due to its frequent exposure to infective pathogens, the respiratory tract is a promising region for the search of novel AMPs which can effectively be isolated from complex peptide libraries [7,8]. Screening and subsequent separation of antibacterial active fractions enabled the identification of endogenous AMPs from various biological materials of human origin. Peptides modulating viral infections were identified from libraries derived from human hemofiltrate [9–12] or human semen [13,14] using cell-based viral infection assays. Human plasma was the source for the identification of human beta-defensin 1 [15] and human milk for the identification of the antibacterial peptide Casein k(63–117) [16]. Additionally, ß-2 Microglobulin (B2M) was purified from amniotic fluid in a screen for antibacterial peptides [17].

B2M is part of the MHC-I receptor that is present at the surface of nearly all nucleated cells [18]. The processed peptide contains 99 amino acids with a molecular weight of 11.7 kDa and is slightly anionic with a net charge of – 2 [19]. In the clinical context, B2M can be used as a biological marker for the renal function. In addition, increased serum levels of B2M are linked to inflammatory disease [20] and HIV-1 infections [21]. Due to its ability to form amyloid fibrils B2M is responsible for the development of hemodialysis related amyloidosis [22,23]. A physiological function of B2M in the context of host defense is indicated by its antibacterial effect. B2M shows activity against *L. monocytogenes, Staphylococcus aureus, Proteus vulgaris, and Escherichia coli* [17]. Furthermore, the peptide is the precursor of a fragment secreted from human respiratory epithelial cells which serves as chemoattractant for THP-1 monocytes, thus enhancing phagocytosis of *Staphylococcus aureus* [24]. A reported dose-dependent upregulation of B2M mRNA and secretion of B2M upon stimulation of amniotic cells with bacterial lipopolysaccharide further supports the hypothesis of the antibacterial peptide function [17].

As the exploration of the human peptidome proved to be a powerful tool for the identification of novel AMPs, we used this method to search for AMPs in the respiratory tract. Screening a peptide library derived from 20 l of pooled broncho-alveolar-lavage (BAL) fluid, resulted in the identification of a fraction with antimicrobial activity against Gram-positive and Gram-negative bacterial pathogens containing B2M. An increased antibacterial activity of B2M was observed at acidic pH values that are a hallmark for infection and inflammation [25]. Furthermore Sytox green uptake and electron microscopic investigations pointed toward a membrane disruptive action of the peptide. By prediction-based fragmentation of B2M and subsequent determination of bacterial growth inhibition we could locate its antimicrobial activity to the C-terminal part of the peptide. B2M may thus play an important role in innate immunity as an antimicrobial peptide.

## Materials and methods

### Preparation of BAL peptide library

Clinical samples of BAL comprising a total of 20 L were collected and immediately frozen for further processing. Peptide/protein extraction was done by acidification with acetic acid to pH 3, followed by centrifugation at 4.200 rpm, and filtration (0.45 µm) of the supernatant. Further, the filtered BAL was subjected to ultrafiltration (cutoff: 30 kDa) yielding 22 L of a sample enriched in peptides and small proteins. Chromatographic fractionation of the ultrafiltrate sample was performed by using a reversed-phase (PS/DVB) HPLC column Sepax Poly RP300 (Sepax Technologies, Newark DE, USA 260,300–30,025) of dimensions 3 × 25 cm, at a flow rate of 55 mL/min with the gradient program (min/%B): 0/5 5/5 20/25 35/50 50/75 55/0, being A, 0.1% TFA (Merck, 1,082,621,000) in ultrapure water, and B, 0.1% TFA in acetonitrile (J.T.Baker, JT9012-3). Seventy-three reversed-phase chromatographic fractions of 50 ml were collected to constitute the BAL peptide bank, from which 1 mL-aliquots (2%) were lyophilized and used for antimicrobial activity testing. For further purification of active fractions, a reversed-phase C18 HPLC column (1 x 25 cm) (Phenomenex, 00 G-4601-N0) was used at a flow rate of 1.8 mL/min with the gradient program (min/%B): 0/5 60/60 70/80.

This study was approved by the Ethics Committee of Ulm University (file number 324/12). BAL was obtained from patients at the Section Pneumology, Internal Medicine II, University Hospital Ulm, Germany. All subjects gave written informed consent in accordance with the Declaration of Helsinki.

### Mass spectrometry analysis

For intact mass measurement, the sample (either unmodified or carbamidomethylated and digested) was analyzed without any modification. Subsequently, MS/MS measurement was done also after carbamidomethylation and digestion with trypsin for sequencing of the proteolytic fragments. The sample was reduced with 5 mM DTT for 20 min at RT, carbamidomethylated with 50 mM iodoacetamide for 20 min at 37°C, and digested with trypsin (ThermoFisher Scientific, 900,589), at a 1:50 ratio (enzyme:protein) for 16 h at 37°C.

A 15 µL-aliquot of the sample (either unmodified or carbamidomethylated and digested) was measured using an LTQ Orbitrap Velos Pro system (Thermo Fisher Scientific) online coupled to an U3000 RSLCnano (Thermo Fisher Scientific) uPLC as described previously [26] with the following modifications: For separation, a binary gradient consisting of solvent A, 0.1% formic acid (Merck, 5,438,040,250) in LC-MS grade water (Merck, 1,153,334,000), and solvent B, 0.1% formic acid in 86% acetonitrile (Merck, 1,000,304,000) was employed. After loading onto the precolumn, the sample was concentrated and washed in 5% B for 5 min. In a first elution step, the percentage of B was raised from 5 to 15% in 5 min, followed by an increase from 15 to 40% B in 30 min. The column was washed with 95% B for 4 min and re-equilibrated for subsequent analysis with 5% B for 19 min.

For visualization in XCalibur Qual Browser (Thermo Fisher Scientific), the theoretical B2M mass spectrum with respect to the observed charge states was predicted from its sequence (UniProtKB – P61769) and compared to the experimental mass spectrum.

Database searches were performed using PEAKs X+, http://www.bioinfor.com/peaks-studio [27]. For peptide identification, MS/MS spectra were correlated with the UniProt human reference proteome set, www.uniprot.org. Carbamidomethylated cysteine was considered as a fixed modification along with oxidation (M) as a variable modification. False discovery rates were set on the peptide level to 1%. Theoretical average molecular masses were calculated with ProtParam, https://web.expasy.org/protparam/.

### Virtual screening and design of modified B2M fragments

To localize the region in B2M responsible for its antibacterial activity, the amino acid sequence of B2M was subjected to a massive virtual screening of all possible fragments with a sequence length between 10 and 30 residues (2078 peptides in total). Our *in-house* machine-learning-based predictor, ABP-Finder (https://protdcal.zmb.uni-due.de/ABP-Finder/index.php) [28], was used to first identify putative antibacterial peptides (ABP), and to predict whether the bacterial targets for each of these ABP belong to the classes Gram-positive, Gram-negative, or to both types of the Gram staining assay. ABP-Finder was used to score the 2078 peptides derived from B2M. Those fragments with a probability score larger than 0.7 were extracted, resulting in 282 peptides. Subsequently, the CD-Hit program [29,30] was used to cluster the annotated peptides using a sequence identity threshold of 70% related to the peptide in the center of the cluster, which led to 19 clusters. Five clusters were discarded because of their low population (see Table S1 in Supplementary Information). From each of the remaining 14 clusters, the peptide with maximum ABP-Finder score was selected. Among these 14 candidates, four peptides were not further considered because their sequences fully overlapped with part of the sequences of other candidates. Thus, the sequence identity screening resulted in 10 representative peptides from the high-scored fragments predicted using ABP-Finder (Table S1). Peptides were then selected for experimental evaluation, based on their scores and the fact that these peptides sampled distinct ranges in the sequence of the B2M peptide (Fig. 7 and Table S1).

Modified fragments of the C-terminal part of B2M containing single amino acid exchanges were generated by AMPA, http://tcoffee.crg.cat/apps/ampa/do [31,32], CAMPR3, http://www.camp.bicnirrh.res.in/prediction.php [33] using the Rational Design of Antimicrobial Peptides tool, and then evaluated by CAMPR3-Predict Antimicrobial Peptide tool, Antibp2, http://crdd.osdd.net/raghava/antibp2/ [34], ClassAMP, http://www.bicnirrh.res.in/classamp/predict.php [35] Peptide AMP Scanner, https://www.dveltri.com/ascan/v2/ascan.html [36] and iAMPpred, http://cabgrid.res.in:8080/amppred/server.php [37].

### Screening the BAL library and purified peptides for antibacterial activity

To identify library fractions and purified peptides with antibacterial activity a radial diffusion assay was carried out. Bacteria were cultured in liquid THY broth (Todd-Hewitt Broth [Oxoid, CM0189B] supplemented with 0.5% yeast extract [BD, Miami, USA, 212,750]) at 37°C overnight in a 5% CO_2_ atmosphere, pelleted by centrifugation and washed in 10 mM sodium phosphate buffer. Following resuspension in 10 mM sodium phosphate buffer optical density was determined spectrophotometrically at 600 nm (OD_600nm_). Bacterial density was adjusted to seed 2 × 10^7^ bacteria into a petri dish in 1% agarose dissolved in 10 mM sodium phosphate buffer. Plates were allowed to cool for 30 min at 4°C before several 2–3 mm holes were placed into the 1% agarose. Freeze-dried fractions of the BAL library pool were reconstituted in 200 µl of ddH_2_O with 10 µl being used for the initial screen. Alternatively, peptides adjusted to the desired concentration in 10 µl of ddH_2_O were filled into the agar-holes. Following incubation at 37°C in ambient air for 3 h plates were overlaid with 10 ml of a 1% agarose solution containing 3% tryptic soy broth (TSB) dissolved in 10 mM phosphate buffer. Inhibition zones in cm were determined following 16–18 h incubation time at 37°C in a 5% CO_2_ atmosphere.

### Survival assay

*L. monocytogenes* cells (ATCC BAA-679/EGD-e) were grown in THY broth at 37°C in a 5% CO_2_ atmosphere overnight. 1 ml of the culture adjusted to an OD_600nm_ of 0.1 was centrifuged and the bacterial pellet was resuspended in 1 ml of assay medium (20% TSB dissolved in 20 mM NaPO_4_ adjusted to different pH values). 90 µl of the bacterial suspension was mixed with 10 µl of the desired concentration of B2M (Lee BioSolutions, St. Louis, Missouri, 126–12-10) or with 10 µl of ddH_2_O (negative control) followed by incubation at 37°C. Samples were taken at the indicated time points, dilutions were prepared and plated on Sheep blood agar plates (TSA+SB, Oxoid, Basingstoke, UK, PB5012A). Colony Forming Units (CFU) were quantified after overnight incubation of the agar plates at 37°C in a 5% atmosphere. Bacterial survival was calculated in comparison to the CFU present at the beginning of the experiment (t = 0 min) and normalized to the mock-treated bacterial sample grown in the corresponding assay medium.

### Sytox green membrane permeabilization

Two different readouts (fluorescence plate reader and flow cytometer) were used to measure Sytox green (Invitrogen, Thermo Fisher Scientific, Dreieich, Germany S7020) uptake into bacterial cells. For samples analyzed by the flow cytometer, an equivalent of 100 µl of a *L. monocytogenes* (ATCC BAA-679/EGD-e) culture grown to an OD_600nm_ of 0.1 were harvested by centrifugation and resuspended in 20% TSB dissolved in 20 mM NaPO_4_ adjusted to three different pH values (pH 7, pH 5.5, pH 4.5). The samples were treated with B2M at a concentration of 1 mg/ml or were mock-treated with ddH_2_O. After incubation at 37°C for 1 h, the cells were pelleted and resuspended in 20% TSB NaPO_4_ adjusted to pH 7, pH 5.5, and pH 4.5 containing 0.02 µM Sytox green stain. Quantification was done by FACS analysis. Bacteria treated with 70% isopropanol served as positive control, mocked treated cells (ddH_2_O) served as negative control. The percentage of Sytox positive cells is depicted by normalizing the analyzed samples to the positive control. *L. monocytogenes* cells analyzed by fluorescence plate reader were grown till an OD_600nm_ of 0.1 was obtained. Bacterial cells were centrifuged and the pellet was resuspended in one volume of 20% TSB NaPO_4_ adjusted to pH 7, pH 5.5 and pH 4.5 with 0.2 µM Sytox. 90 µl of the bacterial solutions were mixed with 10 µl of B2M at a concentration of 1 mg/ml or 10 µl of H_2_O. Bacteria treated with 70% isopropanol for 5 min were used as positive control. The fluorescence intensity was measured with a Tecan infinite M plate reader using an excitation wavelength of 488 nm. Measurements were performed in three biological replicates with three technical replicates per sample.

### Transmission electron microscopy (TEM)

*L. monocytogenes* cells, grown to mid-exponential phase, were harvested by centrifugation and resuspended in 20% TSB dissolved in 20 mM NaPO_4_ (pH 4.5). Following treatment of 5 × 10^9^ cells with 1 mg/ml B2M for 15 min at 37°C cells were pelleted by centrifugation and fixed with 2.5% glutaraldehyde containing 1% saccharose in phosphate buffer (pH 7.3). Samples were washed 5 times with phosphate buffer and postfixed in 2% aqueous osmium tetroxide. After dehydrating the samples in a graded series of 1-propanol, they were blockstained in 1% uranyl acetate and embedded in Epon. Sections were collected on copper grids, contrasted with 0.3% lead citrate for 1 min and imaged in a Zeiss TEM 109 or in a Jeol TEM 1400.

### Thioflavin T (ThT) measurements

To monitor the presence of amyloid fibrils, a 2.5 mM stock solution of ThT (Sigma-Aldrich, Schnelldorf, Germany, T3516) in PBS was prepared and sterile filtered. 1 µl of this solution was added to 15 µl of the peptide sample (1 mg/ml), then the sample was filled up to 100 µl using 84 µl of PBS and incubated for 10 minutes in the dark, at room temperature. Of each preparation, 50 µl were transferred to one well of a Corning® 96 well black polystyrene microplate (Sigma-Aldrich, CLS3603). Fluorescence intensity scans were performed at an excitation wavelength of 450 nm and an emission at 482 nm, using a Synergy H1 hybrid multi-mode reader (Biotek, Bad Friedrichshall, Germany). All values represent fluorescence intensity derived from duplicates minus background activity derived from possible background fluorescence. Duplicates were expressed as mean ± standard error derived from two independent experiments.

### Antimicrobial activity of fibrils

To determine if fibrils of B2M possess antimicrobial activity, a radial diffusion assay was performed. Fibrils were generated by incubation of B2M of 1 mg/ml in 150 µl of 20% TSB NaPO_4_ adjusted to pH 7 and pH 4.5 for 30 min at 37°C. After centrifugation, the supernatant was collected and the pelleted fibrils were dissolved in 150 µl of the corresponding buffer. The supernatant and the fibril solutions were then assayed in a radial diffusion assay as described above.

## Results

### Antimicrobial screening of BAL peptide library

**Figure 1.**
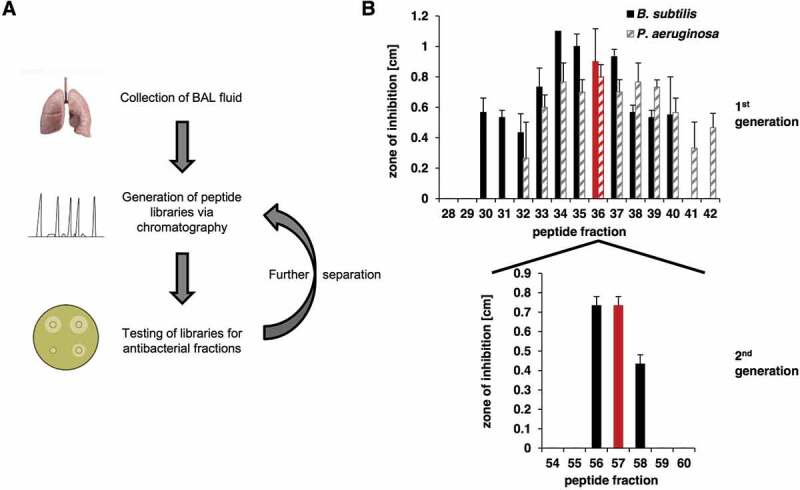
Identification of antimicrobial active BAL fractions. **A**: Illustration of the screening procedure consisting of repeated rounds of peptide separation via chromatography and testing of peptide fractions via radial diffusion assays. **B**: The BAL library was tested in a radial diffusion assay against *B. subtilis* (a Gram-positive species) and *P. aeruginosa* (a Gram-negative species). Peptide fractions 32 to 40 were able to inhibit the growth of both bacteria. The testing of the second peptide library generated from fraction 36 against *B. subtilis* led to three remaining active peptide fractions 56 to 58. Lung: Design created by kjpargeter – www.freepik.com

**Figure 2.**
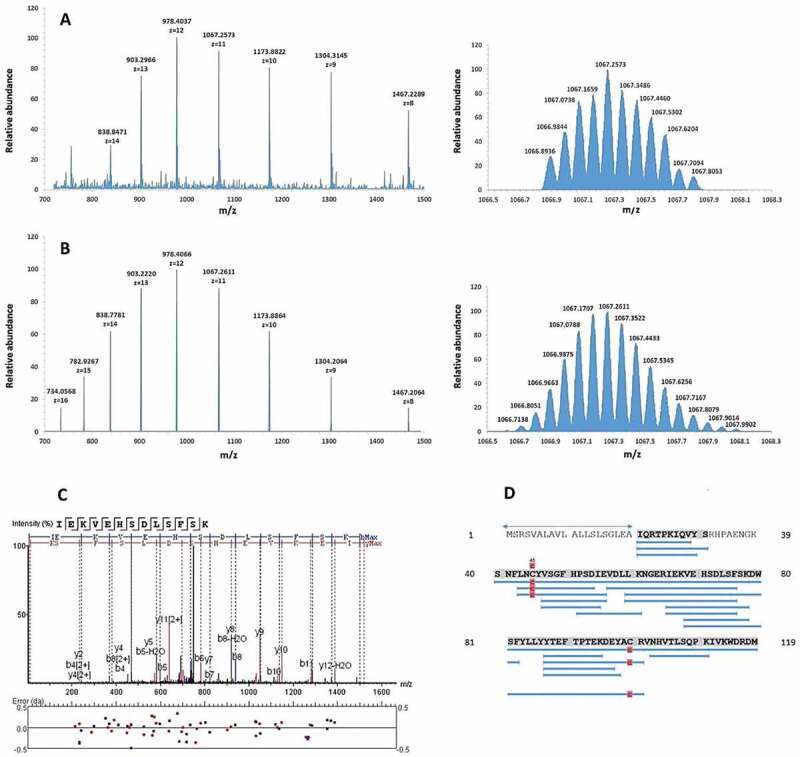
Identification of B2M as antimicrobial active molecule in BAL fluid by mass spectrometry. **A** (left): Electrospray-mass spectrum of antimicrobial fraction 57 showing multiple charged states for B2M. Right: Mass isotope pattern of antimicrobial fraction Fr57 at z = 11. **B** (left): Theoretical multiply-charged ions spectrum of mature B2M (UniprotKB-P61769, 21–119). Right: Theoretical mass isotope pattern of B2M (UniprotKB-P61769, 21–119) at z = 11. Both, the mass/charge state distributions and isotope pattern (z = 11) of fraction 57 (Figure 2(a)) match the respective simulated ones of mature B2M (Figure 2(b)). The calculated average masses are very close: 11,729.41 Da (experimental) vs. 11,729.15 Da (theoretical, from ProtParam). **C**: MS/MS fragmentation spectrum of a B2M proteolytic fragment, corresponding to the sequence IEKVEHSDLSFSK. **D**: Sequence coverage (93%) by sequencing of digested B2M. Delimited blue horizontal bars represent every proteolytic fragment identified in the sequence. Vertical red bars/squares represent a Cys residue identified as carbamidomethylated. Numbers at both sides of the figure represent the residue position in the precursor sequence. The sequence delimited by arrows represents the signal peptide (UniprotKB-P61769, 1–20)

Chromatographic fractionation of the pooled BAL samples yielded 73 fractions that were subsequently screened for inhibiting bacterial growth. For evaluating the antimicrobial activity of the BAL peptide library, a radial diffusion assay that can be carried out with microliter amounts of the fractions of the library was used (Figure 1(a)). Screening was performed with the following bacterial species: *Bacillus subtilis, Pseudomonas aeruginosa, Staphylococcus aureus* MRSA, and *Klebsiella pneumonia*. In the initial screen several neighboring fractions of the library showed a strong antimicrobial activity against *B. subtilis* and *P. aeruginosa*, which were therefore selected for further testing (Figure 1(b)). Fraction 36 was chosen for further analysis due to its location in the middle of the observed peak and based on the fact that it demonstrated the highest antibacterial activity against *P. aeruginosa*, which causes infections in humans. The active fractions were subjected to reversed-phase C18 HPLC and the resulting fractions were evaluated for their antimicrobial activity (Figure 1(b), Fig. S1). A high-intensity chromatographic fraction (Nr. 57) displaying inhibitory activity was separated and analyzed by mass spectrometry. The deconvoluted molecular mass from the dominant multiply-charged ion cluster (Figure 2(a)) was 11,729.41 Da. Subsequent MS/MS analysis of proteolytic fragments generated by tryptic digest of this fraction indicated a 93% coverage of the B2M mature sequence (UniProtKB – P61769, Figure 2 C + D). In addition, the experimental multiply-charged signals closely matched the theoretical ones (Figure 2(b)) as well as the expected molecular mass of 11,729.15 Da, calculated from the mature sequence (21–119) of B2M while considering the formation of a single disulfide bridge. Furthermore, this MS data for B2M is consistent with the MS data from a comprehensive study on human hemofiltrate peptides [38], where mature B2M was identified among others. Taken together, MS data of fraction 57 indicate the presence of mature B2M (21–119).

### Antibacterial activity of B2M

**Figure 3.**
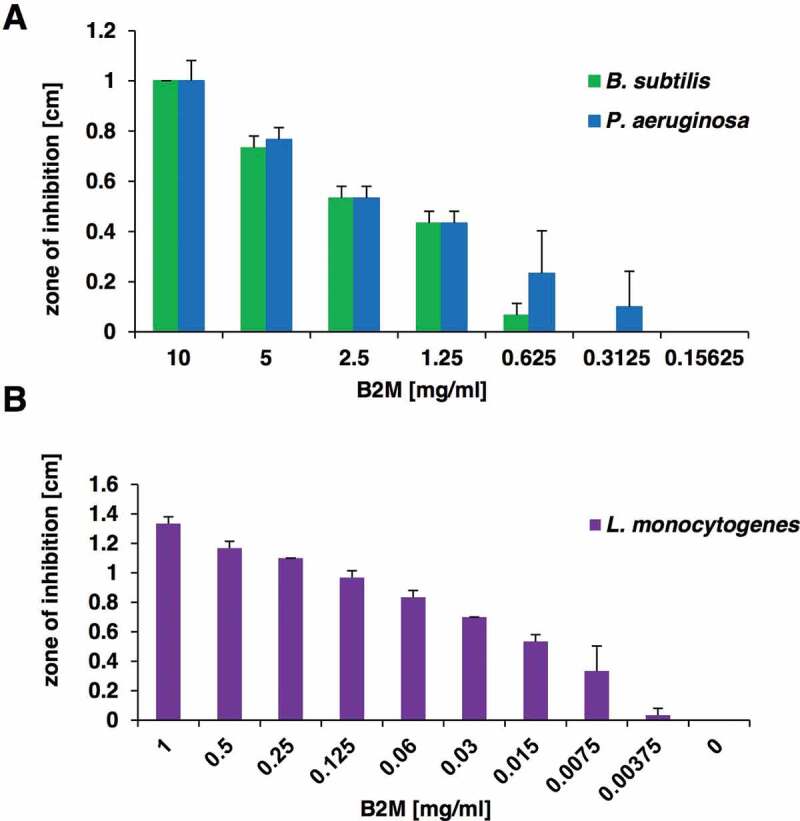
Antibacterial activity of purified B2M. The dose-dependent antibacterial activity of B2M was tested in a radial diffusion assay against the screening strains *B. subtilis* and *P. aeruginosa* (a), and against *L. monocytogenes* (b). The mean value and standard deviation for three independent experiments is depicted

Following the isolation of B2M from the peptide library, purified B2M from a commercial supplier was obtained to investigate its antibacterial activity and thus to substantiate our hypothesis that the antimicrobial effect of the purified peptide library fractions was connected to B2M. For *P. aeruginosa* (ATCC 27853) and *Bacillus subtilis*, a clear dose-dependent growth inhibition could be observed upon exposure to different amounts of B2M (Figure 3(a)). Since a previous publications described an antimicrobial effect of B2M on *L. monocytogenes* [17] the strain ATCC BAA-679/EGD-e was included in our analysis. We confirmed the direct antimicrobial activity of B2M against this strain and show a dose-dependent growth inhibition up to a concentration of 7.5 mg/l (Figure 3(b)).

### pH-dependent antimicrobial effect

**Figure 4.**
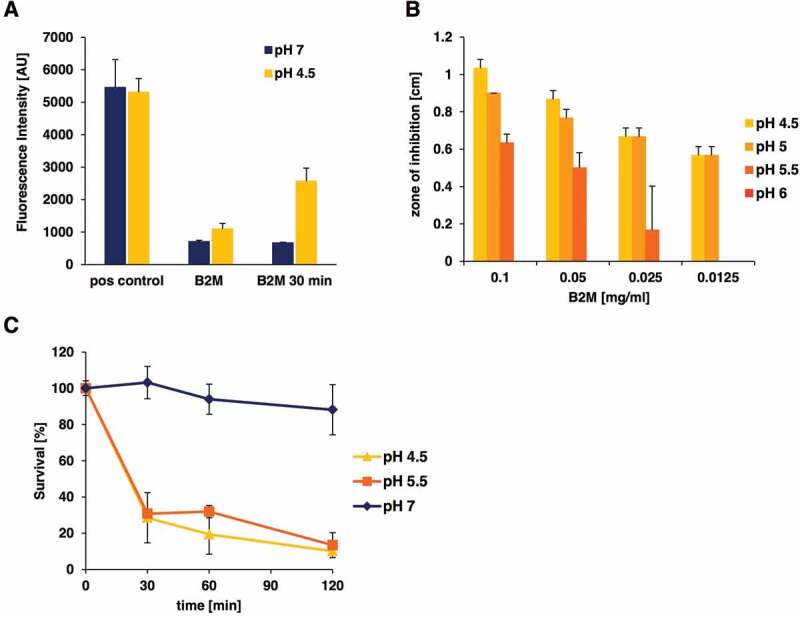
pH-dependent antibacterial activity of purified B2M against *L. monocytogenes*. **A**: Amyloidogenic potential of B2M at low pH conditions. The presence of amyloid fibrils was monitored by ThT staining. B2M was either subjected directly to ThT measurement or was incubated at 37°C for 30 min in buffer with the indicated pH. EF-C fibrils served as positive control (pos control). The mean value and standard deviation for two independent experiments is depicted. **B**: Antibacterial activity of B2M was tested in radial diffusion assays using pH modified agarose solutions (4.5 to 7). Antibacterial activity of B2M in radial diffusion assays could only be observed at pH values lower than 6. **C**: The pH-dependent effect of B2M (1 mg/ml) against *L. monocytogenes* in a survival assay. Peptide and mock-treated bacteria were incubated at pH values 7, 5.5 and 4.5 for 30, 60 and 120 min. Quantification of living bacteria was performed by CFU determination and the values are expressed as % survival compared to mock-treated samples. The mean value ± standard deviation is depicted for three independent experiments

B2M is an amyloidogenic protein known to form fibrils under specific conditions [39]. To investigate, if the antimicrobial effect of B2M is exerted by fibrils or monomers of B2M, thioflavin T measurements of the B2M working solutions used in the assays were carried out and compared to the formation of artificial EF-C fibrils [40] as a control. This analysis revealed (Figure 4(a)) that the B2M solution that was used in our radial diffusion assays, as well as in survival assays is present in its non-aggregated form at pH 7 and shows fibril formation at pH 4.5. After 30 min at pH 4.5 fibrils of B2M could be detected in the solutions that were used for the quantification of antimicrobial activity against *L. monocytogenes* and *P. aeruginosa*. Since we observed fibril formation of B2M at low pH values, bacterial growth inhibition through B2M was also studied under different pH conditions. These investigations were carried out with *L. monocytogenes* and showed a strong pH dependency of the observed antimicrobial activity. In the radial diffusion assays no antimicrobial activity could be detected at pH values of 6 and above, while inhibition zones exceeding 1 cm in diameter were easily visible at pH 4.5 (Figure 4(b)). Similar results were obtained in survival assays, were after 2 hours only about 10% of bacteria were still viable at pH 4.5 and 5.5, whereas identical samples incubated with B2M at pH 7 showed a 90% survival rate (Figure 4(c)). To test if fibrils are involved in the antimicrobial activity of B2M, we assayed the activity of dissolved fibrils in a radial diffusion assay against *L. monocytogenes* (Table 1). Although a visible pellet of mature B2M fibrils could be obtained by incubation of B2M in assay buffer of pH 4.5, no antimicrobial activity of the fibrils present in the pellet could be detected. Only the supernatant of the B2M solution containing monomers, oligomers, and possibly small fragmented fibrils resulted in a visible zone of inhibition.

**Table 1.** Radial diffusion assay of B2M fibril formulations. B2M (1 mg/ml) was incubated for 30 min at 37°C in 150 µl of assay buffer of pH 4.5 or pH 7 allowing for fibril formation. After centrifugation, the supernatant was collected and the resulting fibril pellet was dissolved in 150 µl of assay buffer of pH 4.5 or pH 7. Depicted are the mean ± standard deviation of the zone of inhibition [cm] in a radial diffusion assay against *L. monocytogenes.*

	pH 4.5		pH 7	
	supernatant	fibrils	supernatant	fibrils*
Undiluted	1.03 ± 0.05	0	0.27 ± 0.05	0
1:2	0.90 ± 0.00	0	0	0
1:4	0.83 ± 0.05	0	0	0
1:8	0.63 ± 0.05	0	0	0
1:16	0.43 ± 0.05	0	0	0
B2M 1 mg/ml	1.33 ± 0.05			
B2M 125 µg/ml	1.03 ± 0.05			

* no visible fibril pellet was obtained after centrifugation

### Membrane damage by B2M

**Figure 5.**
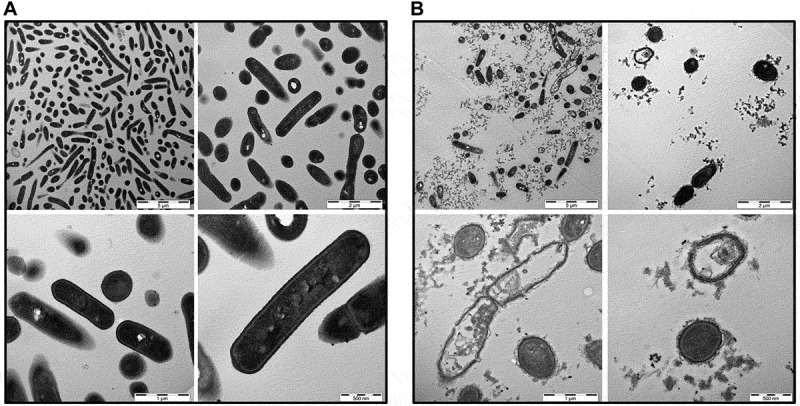
TEM pictures of *L. monocytogenes*. Bacterial cells were either mock-treated with H_2_O (a) or treated with 1 mg/ml B2M for 15 min (b) followed by fixation. Ultra-thin sections (80 nm) were imaged in a Zeiss TEM 109 or in a Jeol TEM 1400. Scale bars are 5 µm for upper left, 2 µm for upper right, 1 µm for lower left and 500 nm for lower right pictures

To measure membrane integrity upon exposure of *L. monocytogenes* to B2M, Sytox green uptake into bacterial cells was determined. Following the treatment of *L. monocytogenes* with 1 mg/ml of B2M for up to 1 h an increased fluorescence could be observed in comparison with the respective controls (Fig. S2). Especially under low pH conditions at values of pH 5.5 and 4.5 a considerable proportion of bacterial cells showed a positive fluorescence staining of nucleic acids which was already visible after 15 min of peptide treatment. The results indicate that B2M exerts its antimicrobial properties through a direct membrane damaging effect on *L. monocytogenes*. To further investigate the mechanisms of action of B2M and to visualize the putative membrane damaging effect TEM was carried out. *L. monocytogenes* bacterial cells were treated with 1 mg/ml B2M for 15 min and 2 hours (data not shown) at pH 4.5. Subsequent TEM pictures revealed a substantial reduction of intact bacterial cells following the treatment of *L. monocytogenes* for only 15 min (Figure 5(b)) while controls appeared intact (Figure 5(a)). Taken together these results suggest that B2M damages bacterial cells in a direct interaction with bacterial surface structures resulting in a rapid bacterial lysis.

### Localization and optimization of active B2M fragments

**Figure 6.**
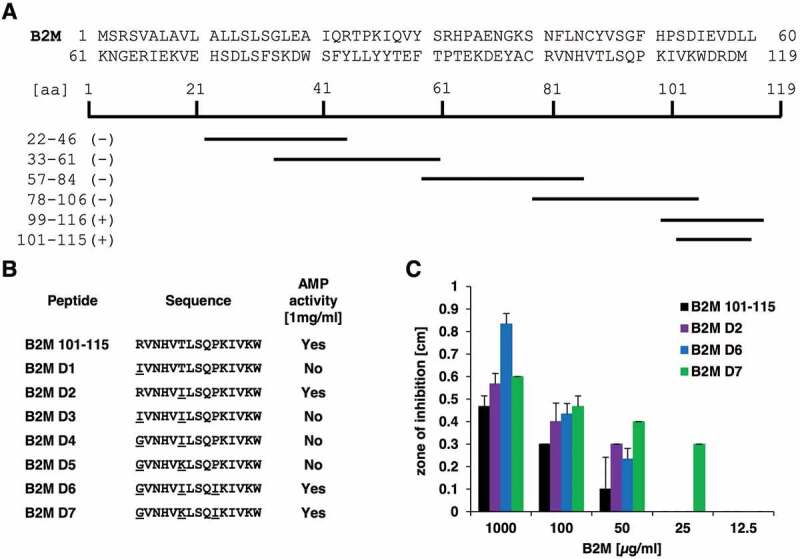
Overview of B2M derivatives and their AMP activity. **A**: Sequence of full-length B2M and the truncated peptides experimentally tested for their antimicrobial activity. Peptides marked with a minus (-) showed no activity against *P. aeruginosa* at a concentration of 1 mg/ml, whereas the fragments located in the C-terminal part of B2M highlighted with a plus (+) were able to inhibit growth of the bacterium in a radial diffusion assay. **B**: Derivatives of the C-terminal part of B2M encompassing amino acids 101 to 115 and their activity against *P. aeruginosa*. All active peptides were tested in triplicates. **C**: Dose-dependent activity of the B2M derivatives tested in a radial diffusion assay. Depicted is the mean ± standard deviation of three independent assays

To further examine, which part of the B2M molecule is responsible for the observed antimicrobial activity, several smaller peptide fragments spanning the entire protein of B2M were generated (Figure 6(a)) based on the virtual screening analysis of B2M for AMPs (Table S1). These peptides, were then tested for antimicrobial activity against *P. aeruginosa* at 1 mg/ml. Results of these assays could locate the antimicrobial activity of B2M to a C-terminal fragment of 18 amino acids (B2M_99-116, ACRVNHVTLSQPKIVKWD). In an effort to optimize the antibacterial effect of this fragment several modified versions of the peptide were synthesized. A shorter variant (B2M 101–115, RVNHVTLSQPKIVKW) was generated by AMPA, an automated webtool, allowing the detection of peptide fragments with putative AMP function in larger proteins [32]. The fragment B2M 101–115 covers most amino acid residues of the entire B2M, which are predicted to exert AMP activity by AMPA. Then, several derivatives (Figure 6(b)) of this fragment were generated by CAMPR3, an online database which contains a sequence optimization algorithm for the rational design of AMPs [33]. The resulting derivatives were evaluated by the programs CAMPR3, antiBP2, ClassAMP, Antimicrobial Peptide Scanner vr.2 and iAMPpred to confirm their intended AMP activity. The derivatives D1-D7 (Figure 6(b)) carry up to three amino acid residues replacements at positions 1, 6 and 10, respectively, which were subsequently tested against *P. aeruginosa*. Among these derivatives, the fragments D6 (GVNHVILSQIKIVKW) and D7 (GVNHVKLSQIKIVKW) showed the most convincing enhancement of antimicrobial activity (Figure 6(c)). Compared to the activity of the modified fragment D7 at 25 µg/ml, 2–4 times the amount of the unmodified 15 residues long fragment B2M 101–115 (50–100 µg/ml) were needed to achieve similar inhibition zones.

## Discussion

Due to a close and constant exposure to the surrounding environment, the respiratory tract represents a major entry portal for microbial pathogens, which may then cause subsequent pulmonary infections. To prevent frequent infections respiratory secretions abundantly contain antimicrobial substances. Already 100 years ago Alexander Fleming studied nasal secretions for antimicrobial activity, which led to the discovery of lysozyme [41]. To explore innate respiratory defense mechanisms we screened a human peptide library originating from bronchoalveolar lavage material for antimicrobial peptides, a screen which led to the identification of B2M in one of the fractions with high antibacterial activity (Figure 1(b)) against *P. aeruginosa* and *B. subtilis*. Specific antimicrobial activity of B2M against *P. aeruginosa, B. subtilis*, and *L. monocytogenes* could be confirmed with commercially available B2M. For *L. monocytogenes* a dose-dependent antibacterial activity could still be detected at a concentration as low as 7.5 mg/l (Figure 3(b)). Our data demonstrate a direct antimicrobial effect of B2M comprising Gram-negative as well as Gram-positive bacterial pathogens.

B2M is a small amyloidogenic human protein of 99 amino acids with a molecular weight of 11.7 kDa. It is found in varying amounts throughout different organs and tissues of the human body, since it is part of the MHC class I complex present on all nucleated cells. It is associated with the MHC class I molecule but not covalently bound to it. B2M is essential for antigen binding and known to play a role in maintaining the correct structure of the MHC class I complex [42]. In BAL fluid of healthy adults, it reaches concentrations of 0.08 mg/l with higher concentrations observed in children [43], while serum concentrations of B2M range between 1 and 3 mg/l [20]. A large fragment of B2M has also been detected in the culture supernatant of respiratory epithelial cells upon stimulation with IL-1ß [24]. It was shown to generate aggregates of *S. aureus*, facilitating the phagocytosis of clusters of bacteria by THP-1 cells. Among various other antimicrobial peptides, B2M has previously been detected in human airway fluid demonstrating antimicrobial activity against *P. aeruginosa* and *L. monocytogenes* [44]. In animal models B2M knock out mice showed enhanced susceptibility toward *L. monocytogenes* [45] as well as *K. pneumonia* infections [46]. However, in animal experiments, it is difficult to decide, which part of the antimicrobial effect may be due to a direct antimicrobial activity of B2M and which part is due to the loss of immunological functions that accompanies a B2M knockout.

Concerning its amyloidogenic nature under pathological conditions, B2M is the main cause of dialysis-associated amyloidosis [47], a property that is specific for human B2M. Human B2M has the ability to form fibrils, in contrast to mouse B2M, which therefore does not represent an amyloid [39]. The antimicrobial properties of amyloids represent a major research focus of recent years. Apart from amyloid beta, which causes Alzheimer’s disease and has been shown to exert antimicrobial properties several years ago [48,49], other amyloid-forming polypeptides such as protegrin-1 serum amyloid A, and temporins, have been demonstrated to inhibit bacterial growth [50]. Additional antimicrobial properties of amyloid ß and protegrin include antiviral and antifungal activities. B2M has previously been associated with antimicrobial activity against *L. monocytogenes* [17], however a detailed analysis of the antimicrobial mechanism and which part of the molecule is responsible for its antibacterial activity has not been performed. The analysis of B2M preparations that were used in antimicrobial studies by thioflavin T assays did not show any mature fibrils at pH 7 but fibril formation occurred at pH 4.5, where a strong antimicrobial effect was observed (Figure 4). To investigate if the antibacterial effect may be linked to the fibrillar form of human B2M, which occurs at low pH [39], the antibacterial activity of B2M fibrils and monomers was studied. Interestingly, the amino acid sequence NHVTLSQ, which is responsible for the amyloid formation of B2M is located in the C-terminal fragment of B2M that showed antimicrobial activity in the fragment analysis. However, only the supernatant of a B2M fibril preparation containing the monomeric and oligomeric form of B2M and possibly smaller fibril fragments demonstrated antibacterial activity in radial diffusion assays. Since the pellet of this preparation, which contains mature B2M fibrils, does not demonstrate antimicrobial activity, these fibrils cannot be responsible for the inhibition zones that are produced by B2M in radial diffusion assays (Figures 3, and 4). A previous publication showed a strong pH-dependent membrane damage of B2M fibril fragments in assays with artificial anionic phospholipid membranes [51]. These results are consistent with our observation under the assumption that small fragmented B2M fibrils were present in the supernatant of our fibril preparation.

Antimicrobial peptides may kill bacteria through various mechanisms. One of the most common mechanisms relies on the disruption of bacterial membrane integrity through the interaction of cationic AMPs with the anionic bacterial plasma membrane present in Gram-negative as well as Gram-positive bacterial cells [52]. To explore the effects of B2M on bacterial cell integrity, Sytox green experiments were performed showing a significant bacterial membrane disruption in a pH-dependent manner as early as 15 min after B2M exposure (Fig. S2). Electron microscopy analysis of bacteria incubated for only 15 minutes with B2M supported the hypothesis of a lytic action as the relevant antimicrobial mechanisms. In addition to a marked reduction of the number of bacteria present in samples treated with B2M, damaged bacterial cells devoid of intracellular content could clearly be detected in the TEM pictures.

The activity of many AMPs is pH-dependent. Quite a number of well-characterized antimicrobial peptides including AMPs like the cathelicidin LL-37, lactoferrin, hepcidin, histatins, dermcidins and the hemoglobin fragment HBB demonstrate a low pH optimum [53,54]. Since acidic pH-values are present at sites of infection, the skin, the vagina, in sweat and within the lysosomal compartment of phagocytic cells, an increased antimicrobial activity under these conditions is advantageous for the host. B2M as an ubiquitous human cell surface molecule is expected to be present in all of this different host niches and has been demonstrated, for example, in sweat [55]. The protonation of histidine, aspartic acid, and glutamic acid residues has been suggested as a relevant mechanisms to explain the altered activity of AMPs at low pH [53]. In our case the protonation of amino acids may have been facilitated by the extraction of peptides from the BAL fluid under acidic conditions. Furthermore, the net charge of B2M changes from −2 at neutral pH to a net positive charge of about 4–5 at pH 5 (http://protcalc.sourceforge.net/). These changes may facilitate the insertion of B2M into anionic bacterial membranes or the formation of the responsible bioactive structure. Our results clearly show a pH-dependent antimicrobial activity for B2M, which is supported by an increase in membrane damage at low pH.

In summary, we were able to isolate B2M as an antimicrobial peptide from the respiratory tract. We could show a pH-dependent antimicrobial activity that can be located to the C-terminus of the molecule. B2M causes a rapid lysis of bacterial cells which appears to be attributable to an interference of B2M with bacterial cell membrane integrity.

## Supplementary Material

Supplemental Material
