# Supplementary material for: Respiratory ß-2-Microglobulin exerts pH dependent antimicrobial activity
Source: Virulence. 2020 Oct 22;11(1):1402–14. doi: 10.1080/21505594.2020.1831367 (PMC7588194; doi:10.1080/21505594.2020.1831367)
Supplement: Supplemental Material [file KVIR_A_1831367_SM2249.zip › B2M-Holch-SupplementaryMaterial.docx]

**Supplementary material**

**Table S1.** Representative peptides extracted from the clustering analysis. The numbers in the fragment IDs correspond to the ranges in the B2M’s sequence. The table is ordered according the positions of the fragments in the sequence of B2M. The table summarizes the ABP-Finder score, as well as the cluster populations and the screening criteria for each peptide. In red are highlighted the final set of peptides selected for experimental evaluation.

| Fragment ID | ABP-Finder Score | Cluster Population (%) | SCREENING |
| --- | --- | --- | --- |
| B2M:1-10 | 0.80 | 0.41 | Low population |
| B2M:22-32 | 0.83 | 1.23 | Low population |
| B2M:22-43 | 0.96 | 8.64 | Overlap |
| B2M:22-46 | **0.98** | **16.46** | OK_Selected |
| B2M:24-43 | 0.93 | 3.70 | Overlap |
| B2M:33-61 | **0.98** | **18.11** | OK_Selected |
| B2M:45-54 | 0.79 | 0.41 | Low population |
| B2M:45-70 | **0.91** | **6.17** | OK |
| B2M:55-64 | **0.97** | **2.47** | OK |
| B2M:56-67 | **0.95** | **4.94** | OK |
| B2M:57-84 | **0.96** | **4.12** | OK_Selected |
| B2M:61-70 | 0.70 | 0.41 | Low population |
| B2M:77-100 | **0.88** | **9.05** | OK |
| B2M:78-106 | **0.92** | **9.88** | OK_Selected |
| B2M:80-89 | 0.90 | 0.41 | Low population |
| B2M:84-99 | 0.85 | 4.94 | Overlap |
| B2M:88-99 | **0.86** | **2.47** | OK |
| B2M:99-116 | **0.92** | **3.70** | OK_Selected |
| B2M:105-114 | 0.90 | 2.47 | Overlap |

Table S2 von Armando, liegt noch als escel Datei vor, muss hier noch eingefügt warden
