# Supplementary material for: Respiratory ß-2-Microglobulin exerts pH dependent antimicrobial activity
Source: Virulence. 2020 Oct 22;11(1):1402–14. doi: 10.1080/21505594.2020.1831367 (PMC7588194; doi:10.1080/21505594.2020.1831367)
Supplement: Supplemental Material [file KVIR_A_1831367_SM2249.zip › Supplementary figure legends.docx]

**Supplementary figure legends**

**Figure S1:** A) Chromatographic fractionation of ultrafiltered BAL by RP (PS/DVB) HPLC. Fraction 36 (32.7-33.6 min) showed antimicrobial activity. B) Chromatographic fractionation of active fraction 36 by RPC18 HPLC. Fractions 56-58 (55-58 min) were active, from which Fr. 57 showed the highest antimicrobial activity and was selected for MS analysis.

**Figure S2**: Effect of B2M on membrane integrity of *L. monocytogenes*. **A:** Time dependent uptake of sytox green measured by fluorescence intensity. Bacteria were either treated with B2M (1 mg/ml) or mock treated with H_2_O (negative control) in buffer at pH 7, 5.5 and 4.5. Isopropanol (70%) treatment served as positive control. Depicted are the mean values and standard deviations of three technical replicates within three biological replicates. Statistical significance was tested for the 15 min values using the Welch two-sample t-test on log-transformed fluorescence intensity values each comparing negative controls and B2M at pH 4.5 (p=0.000725), pH 5.5 (p=0.0003435), and pH 7 (p= 0.2248), p <0.05 is indicated by asterix **B:** Flow cytometric readout to evaluate the percentage of sytox positive cells after B2M treatment. Bacteria were incubated for 1 h with B2M (1 mg/ml) in buffer at pH 7, 5.5 and 4.5 followed by sytox green staining. Bacteria treated with H_2_O served as negative control and isopropanol (70%) treatment was used as positive control. The percentage of sytox positive cells is indicated by normalizing the samples to the positive control. Mean values ± standard deviation of three independent experiments is shown.
