# Supplementary figures and images for: Respiratory ß-2-Microglobulin exerts pH dependent antimicrobial activity
Source: Virulence. 2020 Oct 22;11(1):1402–14. doi: 10.1080/21505594.2020.1831367 (PMC7588194; doi:10.1080/21505594.2020.1831367)

**Figure S1**

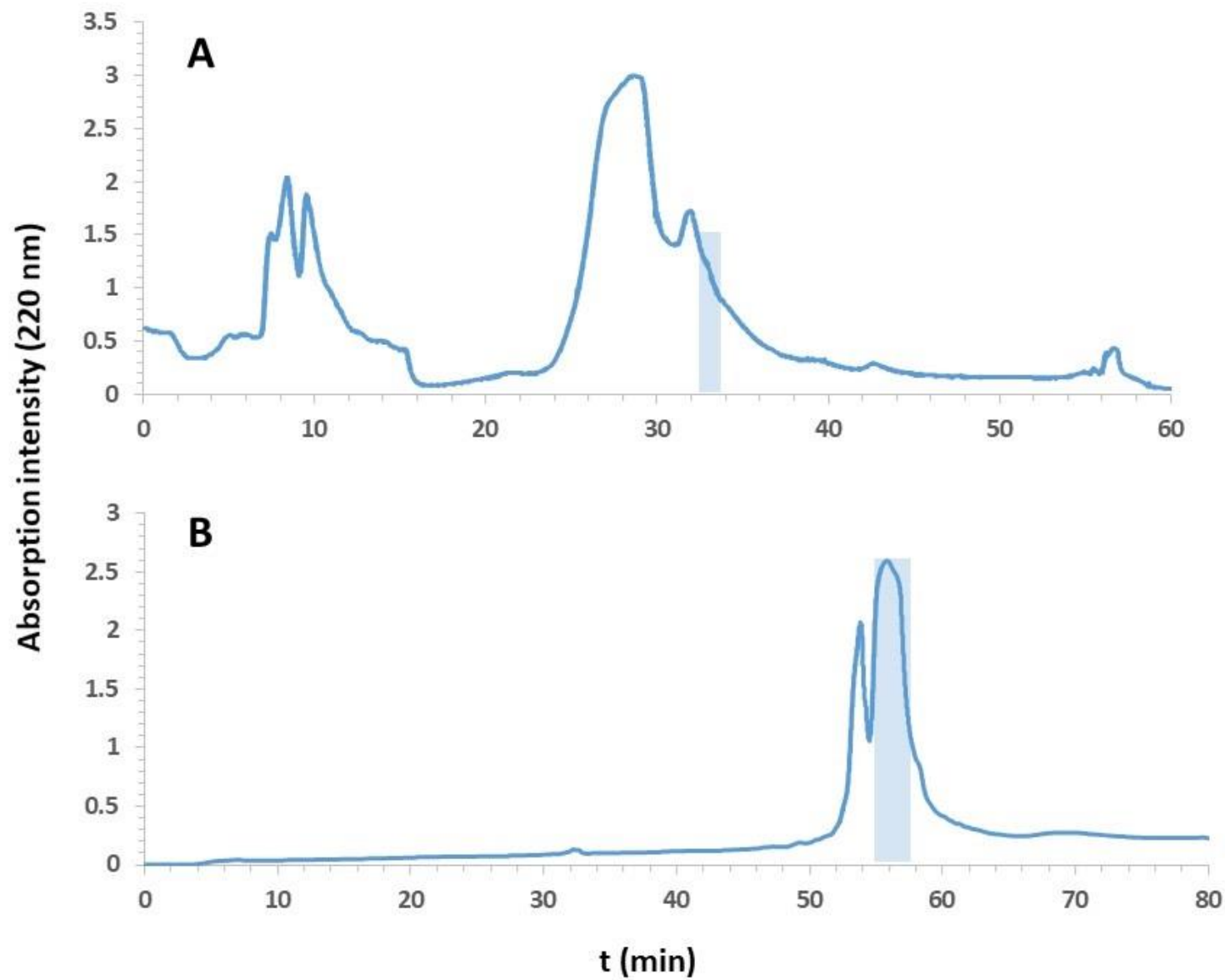

**Figure S2**

**A**

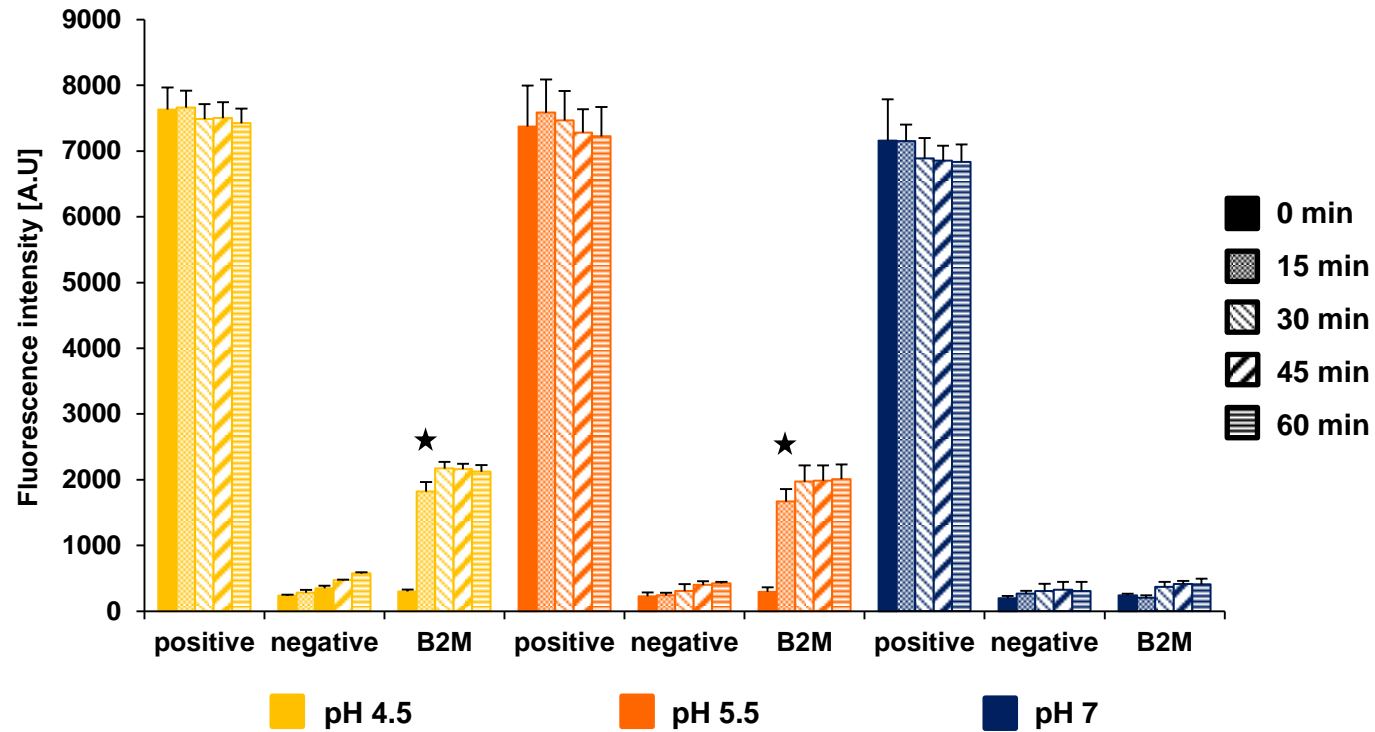

**B**

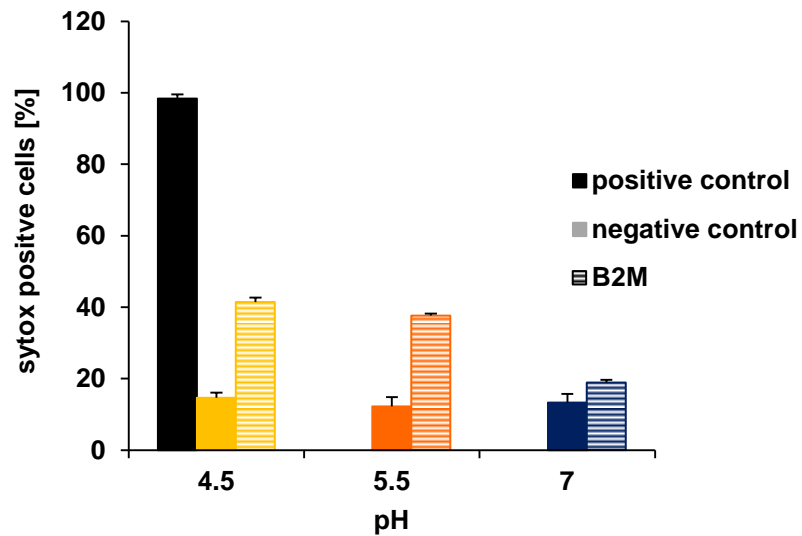

Supplement: Supplemental Material [file KVIR_A_1831367_SM2249.zip › supll figure.pdf]
